# Clinical efficacy of intraoperative real time ultrasound-assisted flexible ureteroscopic holmium laser incision and internal drainage in the treatment of parapelvic cysts

**DOI:** 10.1186/s12893-022-01763-0

**Published:** 2022-08-13

**Authors:** Ting Huang, Qing Yang, Haixiao Wu, Desheng Zhu, Yang Hu, Min Xu

**Affiliations:** Department of Urology, Affiliated Jinhua Hospital, Zhejiang University School of Medicine, Jinhua, 321000 China

**Keywords:** Flexible ureteroscope, Holmium laser, Parapelvic cyst, Ultrasound

## Abstract

**Objective:**

This study aims to investigate the efficacy and safety of intraoperative real time ultrasound-assisted flexible ureteroscopic holmium laser incision and internal drainage in the treatment of parapelvic cysts, and to review recently published relevant literature.

**Method:**

This is a retrospective study in which the clinical data of 47 patients who underwent flexible ureteroscopic holmium laser incision and internal drainage of parapelvic cysts in our center from March 2017 to March 2021 were retrospectively analyzed. A literature search was conducted to review and summarize relevant reports on endoscopic treatment of parapelvic cysts published in the past 10 years.

**Results:**

Among 47 patients with parapelvic cysts who underwent flexible ureteroscopic holmium laser incision and internal drainage, 12 (25.53%) cases had a typical cyst wall bulging into the collecting system under flexible ureteroscope. As the cyst wall was thin and translucent in these cases, ultrasound was not used during the operation. The cysts of the remaining 35 patients were located with the aid of intraoperative real time ultrasound, and all underwent successful operation. No serious surgical complications occurred after surgery. The patients were followed up for 12–24 months after operation. The cyst in one case was observed larger than its original size before operation, so recurrence was considered. In another two cases, the diameters of the cysts were more than half of their original diameters before operation. Thus, the efficacy was poor in the three cases. For the remaining 44 cases, there was no obvious cyst observed or the diameter of the cysts was less than half their preoperative level.

**Conclusion:**

The approach of ultrasound-assisted flexible ureteroscopic holmium laser incision and internal drainage in the treatment of parapelvic cysts is safe and effective, which helps to solve the problem of localization of atypical parapelvic cysts on endoscopic findings.

## Introduction

Renal cyst, a common lesion of kidney, has a population prevalence of approximately 10% [1]. Most renal cysts are simple, asymptomatic, and can be incidentally detected by routine physical examination. Only a small proportion of them require treatment [2]. At present, the treatment of simple renal cysts is quite mature, and there are many choices in terms of operation management. However, parapelvic cyst, as a rare disease, accounts for only a small proportion of renal cysts, and there is no uniform standard for operation management due to the special location and complex adjacent structures. Laparoscopic deroofing is the most effective and minimally invasive method of treating renal cysts. However, the specific anatomical structure of parapelvic cysts increases difficulty in operation and the associated surgical complications. Flexible ureteroscope for treatment of parapelvic cysts is recommended in guideline and has become a growing trend [3]. In recent years, although the use of flexible ureteroscopy in the treatment of renal cystic diseases has become more and more popular, there exists rare relevant literature due to its low prevalence and limited surgery indication. We retrospectively analyzed the clinical data of 47 patients with parapelvic cysts treated with ultrasound-assisted flexible ureteroscopic holmium laser incision and internal drainage from March 2017 to March 2021, and analyzed the safety and efficacy of this procedure and summarized relevant literature published since its application.

## Methods

The clinical data of 47 patients with parapelvic cyst treated with ultrasound-assisted flexible ureteroscopic holmium laser incision and internal drainage in our center from March 2017 to March 2021 were retrospectively analyzed, including preoperative data (general information of patients, location and size of cysts, whether accompanied by stones, etc.), intraoperative data (operation time, whether the cyst performance was typical, etc.), postoperative data and follow-up (postoperative hospital stay, complications, cyst recurrence, etc.) and statistical analysis.

Inclusion criteria were as follows: (1) maximal diameter of renal cyst > 4 cm; (2) symptoms such as lumbago, hematuria, kidney stones, obvious symptoms of the collecting system caused by a parapelvic cyst; (3) endogenous cysts mainly convex to renal hilus as determined by imaging. Exclusion criteria were as follows: (1) patients with a Bosniak classification on CT imaging of grade III and IV; (2) patients with severe cardiac, hepatic, pulmonary and brain dysfunction, without tolerance to general anesthesia; (3) patients thought to have a severe urinary tract infection or with ureteral stricture.

Preoperative examinations included laboratory examinations such as blood and urine routines, electrolytes, renal function, coagulation, and routine biochemistry tests and imaging examinations such as urinary ultrasound, urinary CT + enhancement, and excretory phase imaging.

### Surgical methods

**Fig. 1:**
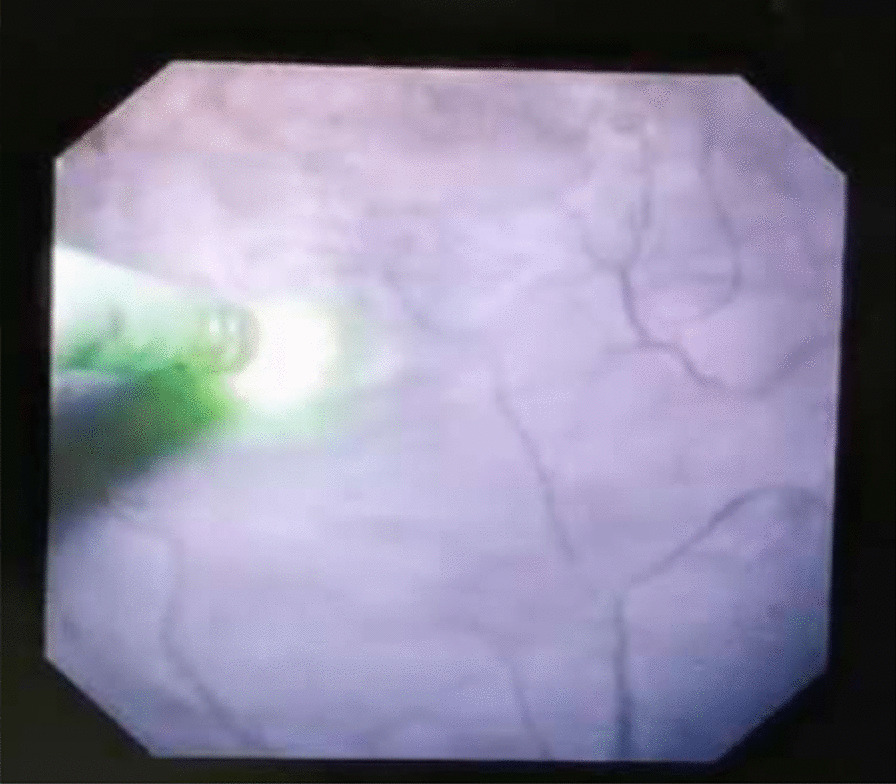
Endoscopic typical findings were darker color of the weak part of the capsule wall and bulging of part of the mucosa into the renal pelvis

**Fig. 2:**
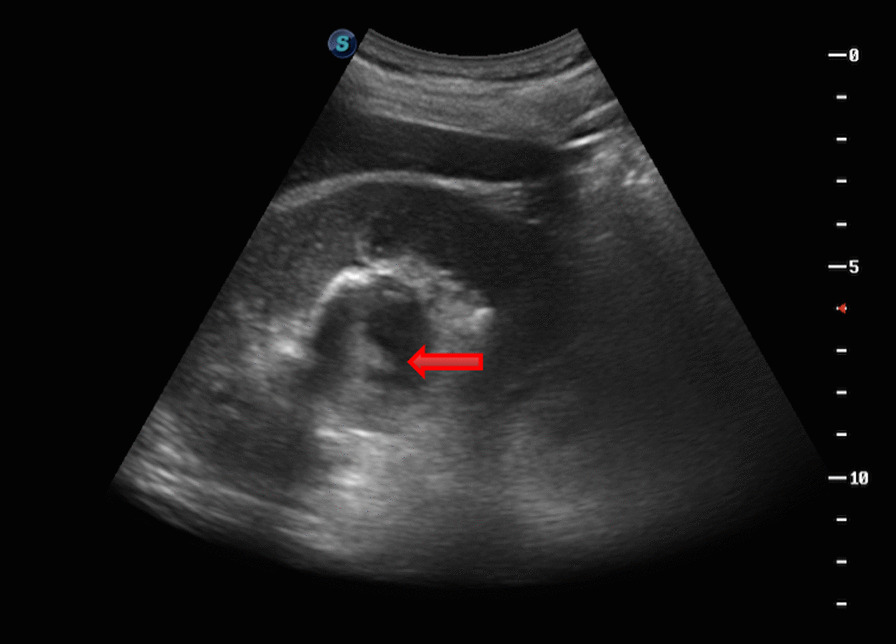
“Smoking” phenomenon was observed when the capsule wall was punctured

Two weeks before surgery, an F6 double-J tube (COOK, USA) was preset to facilitate passage of the ureteroscope, and all patients were treated with the following procedure: general anesthesia, lithotomy position, removal of the preset ureteral stent zebra guide, transurethral insertion of the ureteroscope into the renal pelvis under direct vision, indwelling guide wire and then removal of ureteroscope, placement of an dilator sheath (COOK, USA) to the ureteropelvic junction along the guide wire, placement of a flexible ureteroscope through the dilator sheath, exploration of the renal pelvis and calyces, and identification of the extent of the capsule wall. Typical endoscopic findings were darker color of the weak part of the capsule wall and bulging of part of the mucosa into the renal pelvis (Fig. 1). Patients combined with stones were first treated with holmium laser to break the stones and then remove them with a stone basket. If no typical endoscopic findings were obtained, intraoperative real time ultrasound was applied for precise positioning. With hyperechogenicity shown at the flexible ureteroscope forepart, it was confirmed that the end of the flexible ureteroscope was close to the weakness of the cyst wall beside the renal pelvis. Cyst was drained from the central radial fenestration of the weak site of the cyst wall to the boundary of the weak cyst wall by Holmium laser (power: 60–80 W), then the cyst could be communicated with the collecting system. Usually, under the tension of the cyst wall, the incision of the incised cyst wall showed a state of natural opening. Meanwhile, the blending of pale-yellow cyst fluid and transparent saline used to wash the flexible ureteroscopes visual field was visible. Moreover, the cyst wall was incised until it was about 1 cm away from the renal parenchyma, or the incising would stop when the cyst wall gradually thickened and obvious blood vessel distribution was found. Holmium laser was used for hemostasis when there was bleeding at the incision site. The phenomenon of “smoking” was observed when the capsule wall was punctured (Fig. 2). An F6 double-J tube was indwelled, with its proximal end located in the fenestrated cyst and its distal end located in the bladder. The indwelling catheter was removed 1–3 days after surgery, the double-J tube was removed 1 month after surgery, and the surgical result was reexamined by CT 1 and 12 months after surgery.

### Statistical analysis

IBM SPSS statistics v26.0. software was used to analyze the extracted data. Quantitative variables were compared by using the t test. Continuous data are expressed as the mean and range. Te K-S test was used to check whether the preoperative and postoperative data conformed to a normal distribution, and a t test was used to verify whether the data conformed to a normal distribution. Otherwise, the rank sum test was used. p < 0.05 was defined as a statistically significant difference.

Since BASIRI et al. [4] first used internal incision and drainage of ureteral cyst to treat a case of parapelvic cyst in 2010 and reported the treatment, many scholars have also conducted relevant exploration. By searching PubMed and the Web of Science databases with the term “Parapelvic Cysts”, we found 13 articles on endoscopic surgery related to parapelvic cyst and summarized them.

## Results

**Fig. 3:**
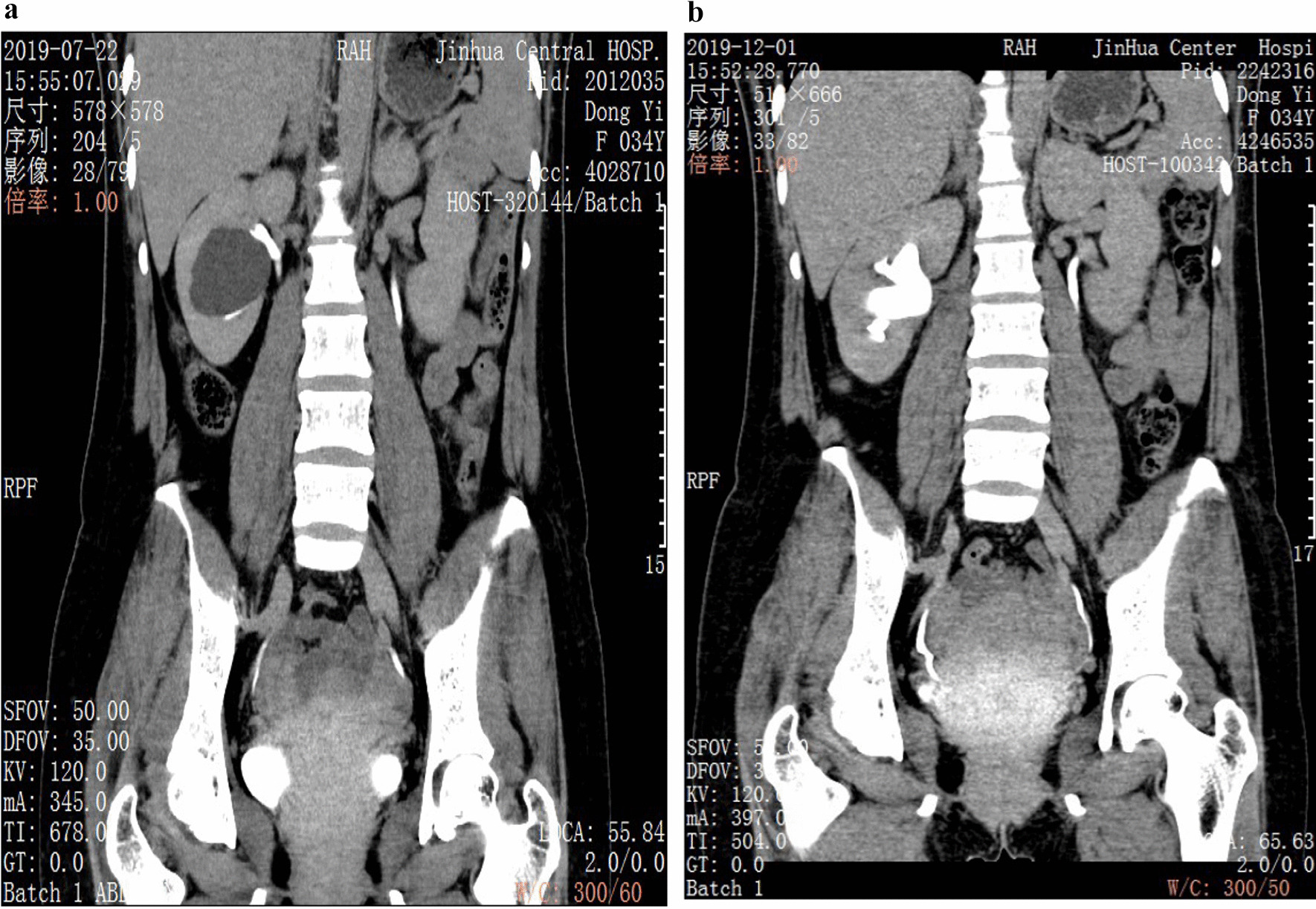
**a** The parapelvic cyst was shown in excretory phase imaging of preopretive urinary CT. **b** CT reexamined 4 months after surgery, the parapelvic cyst disappeared

The general clinical data of the group of patients, aged 58.45 ± 10.86 years, were collected. The lesions were located on the left side in 26 cases, on the right side in 19 cases, and on both sides in 2 cases (one of the cysts was 1.5 cm in diameter, and the affected side was decided to undergo contralateral surgery). The diameter of preoperative lesions was 4.78 ± 1.02 (3.5–7.8) cm. 17 patients presented with lumbar and abdominal discomfort, 4 patients had hematuria as symptoms, and 26 patients were found to be asymptomatic during physical examination. There were 2 cases of hydronephrosis caused by the lesion, one case of nephrydrosis and one case of hydronephrosis of the upper calyx. Additionally, 5 patients with ipsilateral renal calculi received concurrent flexible ureteroscopic holmium laser lithotripsy, and postoperative reexamination showed that all achieved stone-clearing effect. A special case of suprarenal paracalyceal cyst with stenosis of the suprarenal calyceal neck was treated with holmium laser incision of the narrow calyceal neck, followed by incision of the cyst wall after searching for the cyst. By summarizing the clinical data of 47 patients (Table 1), it was found that 6 patients had postoperative lumbar and abdominal pain and 4 had significant gross hematuria, all of which were Clavien-Dindo grade I postoperative complications [5] and were relieved after symptomatic treatment with drugs. All patients were successfully discharged 1–3 days after operation. During a follow-up of 12–24 months after operation, 1 cyst was larger than its original size before operation, and recurrence was considered. Meanwhile, the diameters of the cysts in two cases were more than half of their original diameters before operation. Poor efficacy was considered for the three cases [6, 7]. The cysts of the remaining 44 patients showed no obvious cyst or the cysts were reduced to less than half of their original size before operation (Fig. 3a and b). The maximum diameter of the cysts in 47 patients before and after surgery was compared, which showed a statistical difference (t = 19.631, p < 0.01); diameter variation of the cysts post operation between the maximum diameter of cysts larger (n = 38) than 4 cm and that less (n = 9) than 4 cm was compared, and there was a statistical difference (t = 19.631, p < 0.01).

**Table 1:** Summary of patients’ characteristics

Variable	Data
Patients (n)	47
age (years)	58.45 ± 10.86(34–76)
Largest cyst size (cm) (before surgery)	4.78 ± 1.02(3.5–7.8)
Largest cyst size (cm) (after surgery)	0.72 + 1.25(0–5.0)
Location (n, %)	
Left	26 (55.32%)
Right	19 (40.43%)
Bilateral	2 (4.26%)
Presentation (n, %)	
Upper pole	6 (12.77%)
Medium pole	32 (68.09%)
Lower pole	7 (14.89%)
Mix	2 (4.26%)
Combine with renal stone (n, %)	5 (10.64%)
Symptoms before operation (n, %)	
Abdominal or flank pain	17 (36.17%)
Hematuria	4 (8.51%)
Intraoperative typical performance* (n, %)	12 (25.53%)
Symptoms after operation (n, %)	
Abdominal or flank pain	6(12.77%)
Hematuria	4(8.51%)
Transient fever	8(17.02%)
Operation time	44.70 + 21.00 (15–131)
Mean hospital stay after operation (d)	1.91 + 0.72 (1–3)
Radiologic failure	
1 month after operation (n, %)	1 (2.13%)
12 month after operation (n, %)	3 (6.38%)

*The cyst wall typically was bulging into the collecting system, appearing thin and semitransparent

A total of 13 articles (except for those about laparoscopic techniques) on surgical procedures related to endoscopic parapelvic cysts were identified by searching the databases (Table 2). 11 were retrospective studies, one was prospective study and one did not mention its study category. One study performed percutaneous ureteroscopic laser decortication, one study performed ablation, and the remaining 11 studies performed flexible ureteroscopic laser endopyelotomy, of which five studies used some auxiliary measures (real time ultrasound included) to help locate renal cyst. A total of five cases of serious postoperative complications occurred, including nephrocolic fistula, urogenic urethral stricture and persistent urinary leakage, with a recurrence rate of 0–26.7% [8–20].

**Table 2:** Characteristics of studies

Author (year)	Study type	follow-up duration	No. of patients	Surgical technique	Auxiliary means	No. of patients	recurrent case	Postoperative complications	No. of patients
Kun-Wu (2022) [7]	Retrospective	12 m	17	Flexible ureteroscopic incision	Real time using ultrasound	7	0	Fever	2
								Lumbago	4
Hualin (2021) [8]	Retrospective	9–55 m	33				0		
				Laparoscopy		11		Massive hemorrhage	1
				Flexible ureteroscopic incision		22		Persistent urine leakage	1
Yu (2021) [9]	Retrospective	6 m	90	Flexible ureteroscopic incision			7		
					Holmium laser surgery	43	2	Hematoma	4
					1470-nm diode laser surgery	47	5	Transient fever	10
								Lumbago	17
Baoxing (2020) [10]	Retrospective	6–26 m	31	Flexible ureteroscopic incision	Real time using ultrasound	31	0	0	
Wen (2020) [11]	Retrospective	2 y	28	Flexible ureteroscopic incision		28	4	Transient fever	3
								Hematuria	1
Kang (2018) [12]	Retrospective	15–24 m	11	Flexible ureteroscopic incision			0	Cystitis	2
					Real time using ultrasound	7			
					Percutaneous renal cyst puncture and methylene blue solution w_as injected	3			
					Fiber endoscope was inserted into the parapelvic cavity and operator can see the light from the antegrade fiber endoscope	2			
Wang (2018) [13]	Retrospective	12–36 m	19	Flexible ureteroscopic incision		8	NA	-	
Jia (2017) [14]	Retrospective	3–24 m	6	Percutaneous ureteroscopy laser unroofing	Percutaneous renal cyst puncture and methylene blue solution was injected	11	NA	–	
XiaWa (2015) [15]	Prospective	15 m	21	Flexible ureteroscopic incision		6	0	Urosepsis	1
Zhao (2015) [16]	NA	10–72 m	28	Flexible ureteroscopic incision	Real time using ultrasound	28	1	–	
Weiwen (2015) [17]	Retrospective	12–24 m	35	Flexible ureteroscopic incision		35	0	Fever	1
								Nephrocolic fistula	2
								thrombogenesis in the cystic cavity	3
Luo (2014) [18]	Retrospective	12 m	15	flexible ureteroscopic incision			4	Bladder spasm	3
								Hematuria	1
Korets (2011) [19]	Retrospective	3–6 m	49	Percutaneous Ablation			6	Persistent urine leakage	1
					Bipolar	30			
					Monopolar	19			

NA: data no available

## Discussion

Parapelvic cysts show particularity in terms of location, because they are adjacent to renal pedicle. Compared with common simple renal cysts, parapelvic cysts are more likely to result in compression symptoms of the collecting system or renal pedicle vessels when they are rather small. Therefore, the indications for parapelvic cyst surgery should be appropriately relaxed, and it is not necessary to deliberately follow the treatment indication of simple renal cysts > 5 cm [21] or ≥ 4 cm [22] in diameter, especially for those with symptoms such as hydronephrosis and hematuria should have surgery within a time limit.

At present, there are many means to treat simple renal cysts, including open surgery, puncture and aspiration with or without sclerosing agent, laparoscopic decortication, puncture, retrograde endoscopic drainage and even robot-assisted laparoscopic stripping [1]. However, these treatment methods are not completely applicable to parapelvic cysts. The use of laparoscopic renal cyst decortication in the treatment of parapelvic cysts requires adequate exposure of the renal pedicle. The operation is complex and risky, and most cysts are not significantly convex, so it is difficult to locate them. The use of puncture aspiration in the treatment of parapelvic cysts also faces the risk of causing damage to renal pedicle. It has been reported that the recurrence rate of percutaneous ethanol injection is as high as 72% [23], which can easily lead to ureteropelvic junction obstruction, while the recurrence rate of simple puncture aspiration is even higher. Inverse endoscopic cystotomy seems to be more suitable for the anatomical characteristics of parapelvic cysts, and has the advantages of minimal invasion and repeatability.

Similar to the management of simple renal cysts, relevant examinations and Bosniak classification of cysts should be performed, and the nature of cysts should be systematically assessed. Imaging examination of the urinary excretory phase is helpful to differentiate parapelvic cysts from hydronephrosis [24]. The aforementioned preoperative examinations are necessary to diagnose the disease. For example, Sabrina H Rossi et al. [25] reported that a case of parapelvic cyst with hydronephrosis was misdiagnosed as simple hydronephrosis, in which the patient underwent ureteral stenting but the symptoms recurred several months after stent removal. Ruslan Korets et al. [20] reported a case of persistent urinary leakage after calyceal ablation. All patients included in this study completed preoperative examinations including urinary CT + enhanced and excretory phase imaging. The key to the treatment of parapelvic cysts with flexible ureteroscopic holmium laser incision and internal drainage is to identify the location of cyst and the optimal entry point. However, only a small proportion of the cysts will have a cyst wall bulging into the collecting system under endoscope, showing a typical thin and translucent performance. Most parapelvic cysts are difficult to locate by the naked eye under endoscope. Therefore, intraoperative real-time ultrasound is used to assist in locating the optimal entry point of the cyst. At present, 11 reports have elaborated the localization of parapelvic cysts under intraoperative real-time ultrasound-assisted flexible ureteroscopy. In this study, 12 cases (25.53%) had typical performance under endoscope, and the optimal entry points of the cysts of the remaining 35 cases were located under ultrasound-assisted and the operations were successfully completed.

According to relevant literature, the significance of the technology of intraoperative real-time ultrasound assisted flexible ureteroscope for localization of parapelvic renal cysts has been affirmed by most of the scholars. However, for the current retrospective studies of small sample size, the recurrence rate of cyst post operation varies widely (0–26.7%), which may be related to factors such as different case inclusion criteria and surgical operation criteria. In addition, an interesting finding in the research shows that the patients with a cyst diameter larger than 4 cm may have better benefits. The reason may be that large-diameter cysts allow a wider range of fenestration space during incising process. Therefore, a better communication will be formed. As for the key points of the intraoperative real-time ultrasound assisted flexible ureteroscopes parapelvic cyst open operation, we can make the following conclusions: the range of intraoperative fenestration should be as large as possible, and should be stopped until it is about 1 cm away from the renal parenchyma [26], to avoid uncontrollable bleeding due to the injury. During operation, it is necessary to avoid blood vessel deformation. If a small amount of uncontrolled hemorrhage is found under microscope, for example, holmium laser can be used for hemostasis; after fenestration during operation, the blending phenomenon between the cystic fluid and the transparent saline that is used to wash the flexible ureteroscopes visual field can be observed until the cystic fluid and the washed-down fluid are completely fused due to the fact that the cystic fluid is not completely transparent, most of which is usually pale-yellow. After fenestration, one end of the double-J tube is put into the cyst cavity [27]. One the one hand, the recurrence of the cyst after operation can be avoided; on the other hand, it is helpful to drain the cyst fluid.

The current study has some limitations due to the retrospective nature of the study and the absence of a control group. Considering the low incidence of the disease and limitation of single-center studies on the subjects, it is necessary to conduct multicenter, large sample and longer follow-up studies to further verify our conclusions in the future.

As mentioned above, it can be found that intraoperative real-time ultrasound assisted flexible ureteroscopic holmium laser incision and internal drainage in the treatment of renal cystic diseases have some advantages, such as safe and reliable efficacy, less surgical injury, faster postoperative recovery after surgery and less complications in the perioperative period, especially the better outcomes for the patients with cyst diameter greater than 4 cm, which can be found in the combined short-term studies of small sample size according to the literature reported in recent years and the practice research of the cases. The technology of intraoperative real-time ultrasound assisted flexible ureteroscope for localization of parapelvic renal cysts contributes to treating atypical parapelvic renal cysts accurately with flexible ureteroscopes and avoids iatrogenic injury caused by blind incision. Therefore, the clinical surgical therapeutic effect for patients is good with low cyst recurrence rate in the short term. However, it still needs further study, observation and follow-up based on large sample data to investigate the long-term efficacy and complication prevalence of the operation.

## Data Availability

The datasets used during this study available from the corresponding author on reasonable request.
